# No Impact of Vitamin D on the CYP3A Biomarker 4β-Hydroxycholesterol in Patients with Abnormal Glucose Regulation

**DOI:** 10.1371/journal.pone.0121984

**Published:** 2015-04-02

**Authors:** Buster Mannheimer, Henrik Wagner, Claes-Göran Östenson, Ulf Diczfalusy

**Affiliations:** 1 Karolinska Institutet, Department of Clinical Science and Education at Södersjukhuset, SE 118 82, Stockholm, Sweden; 2 Karolinska Institutet, Department of Molecular Medicine and Surgery, D2:04, SE 171 76, Stockholm, Sweden; 3 Department of Endocrinology, Metabolism and Diabetes at Karolinska Institutet, Karolinska University Hospital, D2:04, SE 171 76, Stockholm, Sweden; 4 Department of Laboratory Medicine, Division of Clinical Chemistry at Karolinska Institutet, Karolinska University Hospital, C1.74, Huddinge, SE-141 86, Stockholm, Sweden; Indiana University Richard M. Fairbanks School of Public Health, UNITED STATES

## Abstract

**Purpose:**

To investigate the effect of vitamin D3 on hepatic Cytochrome P450 enzyme (CYP) 3A4 in patients with abnormal glucose regulation using the endogenous marker 4β-hydroxycholesterol (4β-OHC):cholesterol ratio.

**Methods:**

The present study took advantage of a trial primarily aiming to investigate the effect of vitamin D3 on beta cell function and insulin sensitivity in patients with abnormal glucose regulation. 44 subjects were randomized to receive vitamin D3, 30000 IU given orally once weekly or placebo for 8 weeks. The two sample t-test was used to test the means of the intra-individual differences of 4β-OHC:cholesterol ratio between the two groups.

**Results:**

Mean (SD) 4β-OHC in the whole group of patients before and after the intervention was 26 (11) ng/ml and 26 (12). Mean (SD) 4β-OHC:cholesterol ratio in the whole group of patients before and after the intervention was 0.12 (0.046) and 0.13 (0.047). In the Vitamin D group mean (SD) serum 25-OH-vitamin D3 increased from 46 (16) to 85nM (13) during the corresponding time period. To investigate the impact of vitamin D3 on hepatic CYP3A4 we calculated the mean intra-individual differences in 4β-OHC:cholesterol ratio (delta 4β-OHC:cholesterol ratio) before versus after the intervention in the two treatment groups. The difference (95% CI) between delta 4β-OHC:cholesterol ratio in the control group and intervention group was -0.0010 (-0.0093, 0.0072), a difference being not statistically significant (p = 0.80).

**Conclusions:**

We provide further evidence that vitamin D3 may not substantially affect hepatic CYP3A4. This does not exclude the possibility of an impact of intestinal first-pass metabolism of orally administered drugs which should be investigated.

**Trial Registration:**

ClinicalTrials.gov NCT01497132

## Introduction

Cytochrome P450 (CYP) 3A4 (CYP3A4) is the most important human drug-metabolizing enzyme being involved in at least 50% of the most common drugs [8]. Its activity is subject to a large intra- and inter- individual variability. Food-drug and drug-drug interactions may together with genetic factors explain most of this variability [22,36]. The treatment of diabetes mellitus often demands the use of several drugs whose metabolism often depend on CYP3A4 [10,15,16]. An in vitro study has indicated that CYP 3A4 enzymatic activity is decreased in diabetic patients suggesting that drug metabolism may be decreased in this group of patients [7]. Recently, the findings of seasonable differences in plasma concentrations of the immunosuppressants tacrolimus and sirolimus, known to be metabolized by CYP3A4, suggested that UV-light and the resulting impact on individual vitamin D-levels may contribute to this variation. Thus, significantly lower concentration-to-dose ratios were evident during the months of peak vitamin D levels, July–September, compared with December through February [18]. 4β-hydroxycholesterol (4β-OHC) is formed by the hepatic derived metabolism of cholesterol. Its formation is subject to a CYP3A4 dependent metabolism. The serum concentration of 4β-OHC depends on serum levels of cholesterol. Increasing evidence suggests that serum cholesterol standardised 4β-OHC, i.e. 4β-OHC:cholesterol ratio may be used as a sensitive endogenous marker of this enzyme especially to assess induction but also inhibition [6]. The aim of the present study was to investigate the effect of vitamin D3 on hepatic CYP3A4 using the endogenous marker 4β-OHC:cholesterol ratio.

## Methods

The protocol and supporting CONSORT checklist as well as Source data for this trial are available as supporting information; see S1 Protocol, S1 CONSORT Checklist and S1 Source Data.

### Ethics Statement

The present placebo controlled trial was approved by the Regional Ethics Committee in Stockholm, Karolinska Institutet. Written informed consent was provided from all participants.

### Design

The present study took advantage of a trial primarily aiming to investigate the effect of vitamin D3 on beta cell function and insulin sensitivity in patients with abnormal glucose regulation. The study was a randomized, parallel groups, double-blind, placebo-controlled trial. The funders did not have any role in study design, data collection or the analysis and interpretation of the data.

### Participants

Inclusion criteria were patients with ^1/^ informed consent obtained before any trial-related activities, ^2/^ either an impaired fasting glucose (fasting p-glucose 6.1–6.9 mmol/l), impaired glucose tolerance test (OGTT 2 hour p-glucose 7.8–11.0 mmol/l) or having diabetes (fasting p-glucose ≥ 7.0 mmol/l and/or 2 hour p-glucose ≥ 11.1 mmol/l), ^3/^ ≥ 45 and ≤ 75 years, ^4/^ a body mass index ≤ 32 kg/m^2^, ^5/^ HbA_1c_ ≤ 7.0%, ^6/^ a fasting plasma glucose < 9 mmol/l and ^7/^ serum 25-OH-vitamin D3 < 75 nmol/l. Major exclusion criteria were ^1/^anticipated change in dose of concomitant medication which may interfere with glucose metabolism, such as systemic corticosteroids, non-selective beta-blockers, mono amine oxidase (MAO) inhibitors and anabolic steroids, ^2/^ treatment with any vitamin D preparation, ^3/^ regular sun-bathing in solarium, ^4/^hypercalcemia at screening, defined as free serum calcium > 1.35 mmol/l, ^5/^ hyperphosphatemia at screening defined as serum phospate > 1.5 mmol/l, ^6/^ sarcoidosis or other granulomatous disease ^7/^ all contraindications to vitamin D treatment^8/^ treatment with phenytoin, barbiturates, rifampicin, isoniazid, cardiac glycosides, orlistat or colestyramin, ^9/^disturbed hepatic function defined as alanine aminotransferase (ALAT) ≥ three times the upper reference limit ^10/^ impaired renal function defined as serum creatinine >133 μmol/L for males and >115 μmol/L for females, ^11/^ cardiac disease defined as ^a/^ unstable angina pectoris ^b/^ myocardial infarction within the last 6 months or ^c/^ congestive heart failure NYHA class III and IV, ^12/^cerebral stroke within the last 6 months, ^13/^ uncontrolled treated/untreated hypertension (systolic blood pressure ≥ 180 mmHg and/or diastolic blood pressure ≥ 110mmHg), ^14/^ cancer (except basal cell skin cancer or squamous cell skin cancer), ^15/^ anti-diabetic medication of any kind, ^16/^ females of childbearing potential (menopause is defined as > 1 year since last menstruation) who are pregnant, breast-feeding or intend to become pregnant or are not using adequate contraceptive methods and ^17/^known or suspected abuse of alcohol or narcotics. Individuals were recruited from the Stockholm Diabetes Prevention Programme (SDPP). The cohort had from 1992 been repeatedly investigated with regard to among other things body measurements and oral glucose tolerance test (OGTT) [9]. Individuals that were judged to meet eligibility criteria were phoned and asked to be screened for possible participation.

### Intervention

The collection of the data as well as the intervention was assessed at the metabolic research laboratories in the Department of Endocrinology, Metabolism and Diabetes at Karolinska University Hospital and Södersjukhuset respectively. The subjects were randomized to receive vitamin D3, 30000 IU given orally once weekly or placebo for 8 weeks. The study drug was given by a study nurse at visits to the study site at study weeks 0 and 4. The subjects administered the study drug at home at weeks 1, 2, 3, 5, 6 and 7. Telephone contacts were carried out at these time points to optimize compliance.

### Outcomes

4β-OHC was measured before and after the intervention and compared between the groups. The primary outcome was intra-individual differences in 4β-OHC:cholesterol ratio between the two groups of patients. In addition we investigated the association between insulin resistance and body mass index (BMI) respectively and 4β-OHC:cholesterol ratio in the whole study group before and after the intervention. BMI was defined as mass (kg) /(height (m))^2^. 4β-OHC was determined by isotope dilution gas chromatography-mass spectrometry using deuterium labelled [^2^H_6_] 4β-OHC as internal standard, as described previously [7,11]. Insulin sensitivity was assessed by the hyperglycaemic clamp. Thus glucose infusion was administered at a variable rate to increase the plasma glucose to a target value 6.9 mmol/l above the fasting glucose value. Blood samples for glucose and insulin were taken every 2 minutes from minute 0 to 14 and every 5–10 minutes from minute 14 to 120. The ratio of the glucose infusion rate and plasma insulin concentration (M/I) during the last 30 minutes of the investigation assessed insulin sensitivity expressed in mg/(kg X min) per μU/ml X 100 [4].

### Randomization

Randomization was carried out in a 1:1 manner and administered by the Investigator. The treatment assignment was double-blind. A randomization list was prepared by a statistician not involved in the study. The list contained the randomization numbers 1 to 44 and corresponding information on study medication (active substance or placebo). The study medication was marked with a randomization number, 1 to 44, by Apotek Produktion & Laboratorier AB (APL), according to the randomization list. Subjects that were included in the study were sequentially assigned a randomization number, and thereby the corresponding study medication.

### Adherence rate

The first and fifth doses were administered by the study nurse at the randomization visit, and half-time visit respectively. The remaining six doses were self-administered at home by the study participant. A telephone contact was carried out each week to optimize compliance. Study drug bottles by each participant were brought to the study center at the half-time visit and at the last visit to measure remaining study drug (´pill-count´). No deviations in the retrieved study drugs were recorded, and compliance was therefore estimated to 100%.

### Analysis

The two sample t-test was used to test the means of the intra-individual differences of 4β-OHC:cholesterol ratio between the two groups. The intraindividual differences of 4β-OHC:cholesterol ratio were fairly normally distributed with a skewness of 0.49 which justified the use of untransformed values.

Univariate and multiple linear regression analysis was performed to investigate the effect of the independent variables insulin resistance and BMI, respectively, on the plasma levels of 4β-OHC:cholesterol ratio. Prior to the t-test and regression analysis, 4β-OHC:cholesterol ratio was log-transformed (base 10) in order to achieve a normal distribution. No other factors than the ones described in the variables section were adjusted for.

To test the assumptions for the two linear regression models respective independent variable BMI and insulin sensitivity was plotted against the dependent variable. Both relationships were crudely linear. Standardised residuals were plotted against standardised predicted values to test statistical independence and constance variance of the errors. No apparent deviations were noted. According to Kolmogorov-Smirnov, no significant deviation from the normality of the error distribution was noted (p = 0.20 and p = 0.20 respectively). To identify cases with unusual combinations of values for the independent variables and cases that may have a large impact on the regression model and to study influencial points, we calculated Cook's distance and DfBeta. Max cook's distance for the two models were 0.52 and 0.74 and max Dfbeta were 0.26 and 0.30 respectively.

## Results

**Fig 1:**
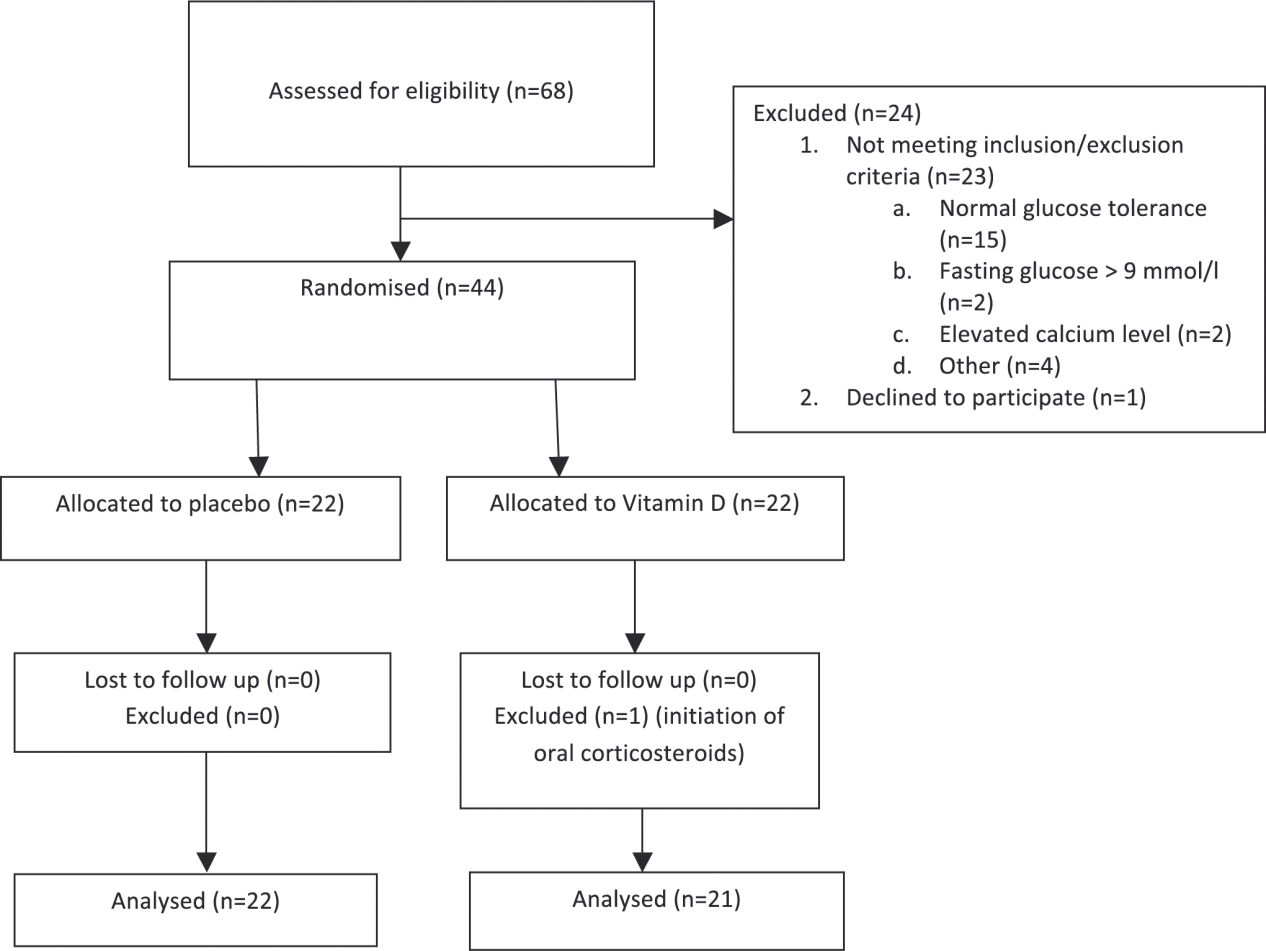
Patient flow chart.

The individuals were recruited from February 29, 2012 to March 15, 2013 and subsequently followed up from March 2, 2012 to May 29, 2013. Forty-four patients were included, 22 patients in the control group and 22 patients in the Vitamin D group. The baseline characteristics of the groups were similar (Table 1). One 67 year old female in Vitamin D group was excluded after randomization due to the initiation of oral corticosteroids due to relapse in her chronic inflammatory bowel disease. Thus 22 patients in the control and 21 in the Vitamin D group were further analysed (Fig 1).

**Table 1:** Baseline characteristics of the study population (n = 44).

	**Control group**	**Vitamin D group**
N females (%)	11 (50%)	10 (45%)
Age (mean±SD^1^) years	66±3.0	66±3.7
Body mass index (kg/m^2^) (mean ±SD)	28 ±2.3	27 ±3.2
4 β -hydroxycholesterol, ng/ml (mean±SD)	25 ± 9	26±13
4 β -hydroxycholesterol:cholesterol ratio (mean±SD)*10^4^	0.12 (0.019)	0.13 ±0.063
Insulin sensitivity M/I^**2**^ (mean±SD)	5.0 ±2.5	6.1±4.2

^1^Standard deviation

^2^Insulin Sensitivity assessed as the ratio of the glucose infusion rate and plasma insulin concentration in mg/(kg X min) per μU/ml X 100

Mean (SD) 4β-OHC in the whole group of patients before and after the intervention was 26 (11) ng/ml and 26 (12). The corresponding mean (SD) 4β-OHC:cholesterol ratio was 0.12 (0.046) and 0.13 (0.047). In the control group mean (SD) serum 25-OH-vitamin D3 was similar before and after the intervention (46 (12) vs 46nM (12)). In the Vitamin D group mean (SD) serum 25-OH-vitamin D3 increased from 46 (16) to 85 (13) nM during the corresponding time period.

To investigate the impact of vitamin D3 on hepatic CYP3A4 we calculated the mean intra-individual differences in the 4β-OHC:cholesterol ratio (delta 4β-OHC:cholesterol ratio) before versus after the intervention in the two treatment groups.

The difference (95% CI) between delta 4β-OHC:cholesterol ratio in the control group and intervention group was -0.0010 (-0.0093, 0.0072), a difference being not statistically significant (p = 0.80).

Univariate and multiple linear regression analysis were performed to investigate the effect of insulin sensitivity and BMI on the plasma levels of 4β-OHC:cholesterol ratio in the whole group of patients.

Insulin sensitivity associated close to significantly (P = 0.064) and significantly (p = 0.008) with 4β-OHC:cholesterol ratio before and after the intervention. However, when BMI was included in the model, the effect of insulin sensitivity vanished (p = 0.89 and p = 0.32) while the effect of BMI remained (p = 0.002 and p = 0.008) (Table 2).

**Table 2:** Univariate and multiple linear regression analysis of insulin sensitivity and body mass index, factors potentially associated with plasma concentrations of 4β-hydroxycholesterol in the study group (n = 43).

**Independent / explanatory variables**	**b** ^1^ **(95% CI** ^2^) **unadjusted**	**p-value unadjusted**	**b (95% CI) adjusted**	**p-value adjusted**
**Before treatment with vitamin D**				
**Insulin sensitivity M/I** ^**3**^	0.01 (-0.001, 0.023)	0.064	0.001 (-0.011, 0.013)	0.89
**Body mass index (kg/m** ^**2**^ **)**	-0.025 (-0.038, -0.013)	<0.001	-0.025 (-0.039, -0.010)	0.002
**After treatment with vitamin D**				
**Insulin sensitivity M/I**	0.014 (0.004, 0.024)	0.008	0.006 (-0.006, 0.017)	0.324
**Body mass index (kg/m** ^**2**^ **)**	-0.023 (-0.035, -0.012)	<0.001	-0.020 (-0.034, -0.006)	0.008

^1^Unstandardised regression coefficient

^2^Confidence Interval

^3^Insulin Sensitivity assessed as the ratio of the glucose infusion rate and plasma insulin concentration in mg/(kg X min) per μU/ml X 100

There were 26 recorded adverse events (AE) in 20 subjects; 10 in the Vitamin D group and 16 in the Placebo group. Intensity was graded as ´severe´ in two AE´s, both fractures of the radius. Otherwise intensity was estimated as ‘mild’ to ‘moderate’. The causality of one AE was judged as probable; diarrhea occurring shortly after the intake of the study drug. The causality for the remaining AEs was judged as unlikely. No serious AE´s occurred during the study.

## Discussion

We investigated the effect of vitamin D on hepatic CYP3A4 using the endogenous marker 4β-OHC:cholesterol ratio in patients with abnormal glucose regulation. We did not see any difference in delta 4β-OHC:cholesterol ratio between patients treated with high dose vitamin D3 and patients that received placebo.

The study was primarily dimensioned to study the effect of vitamin D3 on glucose homeostasis. To get a picture of the strength of our results, a post-hoc calculation was conducted. Thus, with 22 patients in the control and 21 in the Vitamin D group, and a standard deviation of 0.017 we would have 80% power to be able to detect a mean increase in 4β-OHC:cholesterol ratio of 0.015 corresponding to 12%.

This calculation admits the results to be viewed in the light of studies investigating the effect of drugs known to affect CYP3A4. Thus, the initiation of carbamazepin, a drug known to have a large impact of CYP3A4 and often with a significant clinical impact if combined with other drugs whose metabolism being dependent of this enzyme, [26] was studied in 8 paediatric patients with newly diagnosed epilepsy. Carbamazepin was associated with an 800% increase in 4β-OHC [35]. Furthermore, 14 days treatment of rifampicin, also a potent inducer of CYP3A4 [1] in 8 healthy volunteers resulted in a 400% increase in 4β-OHC among the patients that received the highest dose [14]. In 8 male patients with onchomycosis the 1 week treatment with 400mg itraconazole, a potent inbibitor of CYP3A4 [1], resulted in a 29% mean decrease in 4β-OHC[20]. Although being difficult to determine the smallest change in 4β-OHC still being associated with a clinical impact with regard to drugs that are metabolised by CYP3A4, the results of the present study with an effect of less than 12%, seems negligible.

A study using human liver samples obtained from diabetic and nondiabetic donors suggested that diabetes is associated with a significant decrease in hepatic P450 3A4 enzymatic activity and protein level and that CYP3A4 dependent drug metabolism may be decreased in this group of patients [7]. In the present study insulin sensitivity was associated with a decrease in 4β-OHC:cholesterol ratio. However, when including BMI in the model this effect vanished, indicating that insulin resistance per se may not affect plasma levels of 4β-OHC:cholesterol ratio. The results are in accordance with recently published data on kidney transplant patients and may be explained by the increased distribution volume associated with obesity decreasing the plasma levels of 4β-OHC [29]. Furthermore, the mean 4β-OHC concentration in the present study (25 ng/mL) was in accordance with data on four cohorts of volunteers with a normal glucose regulation whose mean ranged from 22–29 ng/mL [3,5].

Importantly, vitamin D does not seem to increase the 4β-OHC:cholesterol ratio. The results are in accordance with a few recent publications. Bjorkhem-Bergman et al. used 6 months data from a clinical trial in 116 patients with antibody deficiency or increased susceptibility to respiratory tract infections who were randomised to either placebo or high-dose (4000 IU/day) vitamin D3 for 12 months. No significant correlation between 4β-OHC and vitamin D levels was observed [2]. Nylén et al. used 2 previously performed studies to study the relationship between plasma levels of vitamin D and 4β-OHC in healthy volunteers from Sweden (n = 45) and Korea (n = 65). No significant correlation was found [21]. The present placebo controlled study has the advantage of using baseline levels 4β-OHC:cholesterol ratio for each individual and thus compare intraindividual differences between the groups. This resulted in a substantially improved statistical power and a study that likely would have been able to detect a 12% increase in 4β-OHC:cholesterol ratio, indeed a relatively small change (see above). No effect was found which provides further evidence that vitamin D does not affect the hepatic drug metabolism of CYP3A4 dependent substrates. However, this does not exclude the possibility of an effect on intestinal first-pass metabolism of orally administered drugs. The expression of CYP3A4 in human duodenal and jejuna mucosal epithelium is known to be high [12,19,23,24,33,34,37] and to be subject to large inter-individual differences. Furthermore, there is considerable data supporting the role of the vitamin D3 (1, 25-dihydroxyvitamin D3) in regulating intestinal CYP3A4 [25,27,28,31,32] while the role for vitamin D receptor in regulating hepatic CYP3A4 is more controversial [11,13,17,37,38]. Investigating human intestinal biopsies Thiumaran et al. showed that intestinal expression of CYP3A4 actually varied seasonally. Midazolam p.o. AUC and oral bioavailability trended higher October through March compared to April through September possibly due to annual changes in UV sunlight and vitamin D levels affecting CYP3A4 activity [30].

### Limitations

Apart from the inherent inability to address intestinally derived CYP3A4 which we do believe is the main limitation, the study sample was rather small, which could have caused a problem of power. However, due to the study design that included measurement of 4β-OHC before and after the intervention, we were able to investigate intraindividual rather than interindividual differences which resulted in rather low standard deviations which substantially increased the power. However, it could be argued that a 12% increase in 4β-OHC might implicate some degree of clinical significance with regard to drugs with small therapeutic windows. Furthermore, the intervention period of 8 weeks could be considered as too short to fully explore the effect of long time exposure of vitamin D3. On the other hand, subjects lost to follow up were minimal and change in lifestyle factors affecting glucose metabolism could also be kept low.

In conclusion, we provide further evidence that vitamin D3 may not substantially affect hepatic CYP3A4. This does not exclude the possibility of an impact of intestinal first-pass metabolism of orally administered drugs which should be investigated.

## Supporting Information

S1 CONSORT Checklist Compliance of the present study to the CONSORT statement. (DOC)

S1 Protocol Effects of vitamin D on beta cell function and insulin sensitivity in pre-diabetes and diabetes mellitus type 2—EVIDENS. (DOC)

S1 Source Data All relevant source data. (XLS)
