# Supplementary material for: No Impact of Vitamin D on the CYP3A Biomarker 4β-Hydroxycholesterol in Patients with Abnormal Glucose Regulation
Source: PLoS One. 2015 Apr 2;10(4):e0121984. doi: 10.1371/journal.pone.0121984 (PMC4383380; doi:10.1371/journal.pone.0121984)
Supplement: S1 Protocol — (DOC) [file pone.0121984.s002.doc]

| ***Effects of vitamin D on beta cell function and insulin sensitivity in pre-diabetes and diabetes mellitus type 2 - EVIDENS*** | | |
| --- | --- | --- |
|  | Products: | Vitamin D3 (Cholecalciferol) |
|  | EudraCT Number: | 2010-024487-18 |
|  | Sponsor: | Claes Göran Östenson, Prof. |
|  | Principal Investigator: | Henrik Wagner, MD |

1. Index

[1 Index 2](#__RefHeading___Toc302319581)

[2 Protocol Summary 4](#__RefHeading___Toc302319582)

[3 Abbreviations 5](#__RefHeading___Toc302319583)

[4 Administrative Information 6](#__RefHeading___Toc302319584)

[5 Background Information 7](#__RefHeading___Toc302319585)

[5.1 Background and Rational 7](#__RefHeading___Toc302319586)

[5.2 Preliminary data 9](#__RefHeading___Toc302319587)

[6 Endpoints 9](#__RefHeading___Toc302319588)

[6.1 Primary Endpoint 9](#__RefHeading___Toc302319589)

[6.2 Secondary Endpoints 9](#__RefHeading___Toc302319590)

[7 Design 10](#__RefHeading___Toc302319591)

[7.1 Outline 10](#__RefHeading___Toc302319592)

[7.2 Visits and telephone contacts 11](#__RefHeading___Toc302319593)

[7.3 Assessments and Procedures 11](#__RefHeading___Toc302319594)

[8 Selection and Withdrawal of Subjects 12](#__RefHeading___Toc302319595)

[8.1 Inclusion Criteria 12](#__RefHeading___Toc302319596)

[8.2 Exclusion Criteria 12](#__RefHeading___Toc302319597)

[8.3 Criteria for Withdrawal 13](#__RefHeading___Toc302319598)

[8.4 Subject Log 13](#__RefHeading___Toc302319599)

[9 Treatment 14](#__RefHeading___Toc302319600)

[9.1 Description of Investigational Medicinal Products 14](#__RefHeading___Toc302319601)

[9.2 Packaging, Labeling, Storage and Handling of Investigational Medicinal Products 14](#__RefHeading___Toc302319602)

[9.3 Treatment Assignment 14](#__RefHeading___Toc302319603)

[9.4 Concomitant Medication 14](#__RefHeading___Toc302319604)

[9.5 Compliance to Treatment 15](#__RefHeading___Toc302319605)

[9.6 Continuation of Treatment 15](#__RefHeading___Toc302319606)

[10 Assessments and procedures 15](#__RefHeading___Toc302319607)

[10.1 Efficacy Assessments 15](#__RefHeading___Toc302319608)

[10.2 Safety Assessments 16](#__RefHeading___Toc302319609)

[10.3 Other assessments 16](#__RefHeading___Toc302319610)

[11 Proceedings for Adverse Events 17](#__RefHeading___Toc302319611)

[11.1 Definition of Adverse Events 17](#__RefHeading___Toc302319612)

[11.2 Assessment of Adverse Events 18](#__RefHeading___Toc302319613)

[11.3 Methods for Eliciting Adverse Events 18](#__RefHeading___Toc302319614)

[11.4 Reporting of Adverse Events 19](#__RefHeading___Toc302319615)

[11.5 Follow-up of Adverse Events 19](#__RefHeading___Toc302319616)

[12 Statistics and Data Management 19](#__RefHeading___Toc302319617)

[12.1 Data Management 19](#__RefHeading___Toc302319618)

[12.2 Statistical Analysis 19](#__RefHeading___Toc302319619)

[12.3 Determination of Sample Size 20](#__RefHeading___Toc302319620)

[13 Direct Access to Source Documents 21](#__RefHeading___Toc302319621)

[14 Quality Control 21](#__RefHeading___Toc302319622)

[14.1 Source Data 21](#__RefHeading___Toc302319623)

[14.2 Monitoring 22](#__RefHeading___Toc302319624)

[15 Ethics 22](#__RefHeading___Toc302319625)

[15.1 Independent Ethics Committee 22](#__RefHeading___Toc302319626)

[15.2 Ethical Conduct of the Trial 23](#__RefHeading___Toc302319627)

[15.3 Subject Information and Informed Consent 23](#__RefHeading___Toc302319628)

[15.4 Risk assessment 23](#__RefHeading___Toc302319629)

[16 Data Handling and Record Keeping 23](#__RefHeading___Toc302319630)

[16.1 Case Report Forms 23](#__RefHeading___Toc302319631)

[16.2 Record Keeping 24](#__RefHeading___Toc302319632)

[17 Insurance 24](#__RefHeading___Toc302319633)

[18 Publication Policy 24](#__RefHeading___Toc302319634)

[19 Supplements 24](#__RefHeading___Toc302319635)

[19.1 Amendments 24](#__RefHeading___Toc302319636)

[19.2 Personnel Information 24](#__RefHeading___Toc302319637)

[20 List of appendices 24](#__RefHeading___Toc302319638)

[20.1 Appendix A: Declaration of Helsinki 24](#__RefHeading___Toc302319639)

[20.2 Appendix B: Schedule of investigational visits 24](#__RefHeading___Toc302319640)

[20.3 Appendix C: IMP labeling 25](#__RefHeading___Toc302319641)

[21 References 25](#__RefHeading___Toc302319642)

[22 Signed Agreement of the Trial Protocol 28](#__RefHeading___Toc302319696)

[23 Appendix A: Declaration of Helsinki 29](#__RefHeading___Toc302319697)

[24 Appendix B: Schedule of investigational visits 34](#__RefHeading___Toc302319698)

[25 Appendix C: IMP labelling 35](#__RefHeading___Toc302319699)

1. Protocol Summary

| **PROTOCOL IDENTITY AND OBJECTIVES** | |
| --- | --- |
| EudraCT Number: |  |
| Protocol Title: | Effects of vitamin D on beta cell function and insulin sensitivity in pre-diabetes and diabetes mellitus type 2 - EVIDENS. |
| Trial Objective: | To evaluate, in persons with IFG, IGT, IFG+IGT or diet treated type 2 diabetes and vitamin D deficiency, the impact of vitamin D3 treatment on beta cell function and insulin sensitivity. |
|  |  |
| **INVESTIGATIONAL MEDICINAL PRODUCTS (IMP)** | |
| Test Product: | Vitamin D3 (Cholecalciferol) 20 000 IU/ml (0.5 mg/ml) |
| Pharmaceutical Form: | Mixture |
| Route of Administration: | Oral |
|  |  |
| **METHODOLOGY** |  |
| Trial Design: | Randomized, parallel groups, double-blind, placebo-controlled trial with 8 weeks intervention period. |
| Dose/Duration: | Vitamin D3 30000 IU weekly, for 8 weeks. |
| Primary Endpoint: | Change in plasma insulin response during hyperglycemic clamp from baseline after 8 weeks. |
| Safety Parameters: | Hypercalcemia, Adverse events |
|  |  |
| **POPULATION OF TRIAL SUBJECTS** |  |
| Description of Trial Subjects: | Subjects with IFG, IGT, IFG+IGT or type 2 diabetes treated with diet. |
| Number of Subjects: | 44 |
|  |  |
| **TRIAL TIMETABLE** |  |
| First Subject In: | Q3 2011 |
| Last Subject In: | Q4 2011 – Q1 2012 |
| Last Subject Out: | Q2 2012 |

1. Abbreviations

| **Abbreviation** | **Explanation** |
| --- | --- |
| AR | Adverse Reaction |
| AE | Adverse Event |
| BMI | Body Mass Index |
| CRF | Case Report Form |
| CVD | Cardiovascular disease |
| FPG | Fasting Plasma Glucose |
| HbA_1c_ | Glycosylated hemoglobin |
| HOMA | Homeostasis Model Assessment |
| HR | Heart Rate |
| IB | Investigator’s Brochure |
| ICH | International Conference of Harmonisation |
| IEC | Independent Ethics Committee |
| IFCC | International Federation of Clinical Chemistry and laboratory medicine |
| IFG | Impaired Fasting Glucose |
| IGF-1 | Insulin-Like Growth Factor-1 |
| IGFBP-1 | Insulin-Like Growth Factor Binding Protein-1 |
| IGT | Impaired Glucose Tolerance |
| IMP | Investigational Medicinal Products |
| ITT | Intention-To-treat |
| IVGTT | Intra-Venous Glucose Tolerance Test |
| GCP | Good Clinical Practice |
| MPA | Medicinal Product Agency |
| OAD | Oral Antidiabetic Drug |
| OGTT | Oral Glucose Tolerance Test |
| PG | Plasma Glucose |
| PP | Per Protocol |
| PPG | Post-prandial Plasma Glucose |
| SAR | Serious Adverse Reaction |
| SAE | Serious Adverse Event |
| SDPP | Stockholm Diabetes Prevention Programme |
| SMPG | Self Measured Plasma Glucose |
| SPC | Summary of Product Characteristics |
| SUSAR | Suspected Unexpected Serious Adverse Reaction |
| T2D | Type 2 Diabetes |
| WC | Waist Circumference |
| 1,25(OH)D | 1,25-dihydroxy-vitamin D3 |
| 25(OH)D | 25-hydroxy-vitamin D3 |

1. Administrative Information

| **Sponsor**  Claes Göran Östenson, Prof.,  Dept. of Endocrinology, Metabolism and Diabetes, Karolinska University Hospital  Phone: +46-8-517 762 00  Fax: +46-8-517 730 96  E-mail: claes-goran.ostenson@ki.se    **Principal Investigator**  Henrik Wagner, MD.  Dept. of Endocrinology, Metabolism and Diabetes, Karolinska University Hospital  171 76 Stockholm, Sweden  Phone: +46-8-517 793 38  Fax: +46-8-517 730 96  E-mail: [henrik.wagner@karolinska.se](mailto:henrik.wagner@karolinska.se)  **Co-Investigators**  Michael Alvarsson, Assoc. Prof.,  Dept. of Endocrinology, Metabolism and Diabetes, Karolinska University Hospital  Phone: +46-8-517 728 62  E-mail: michael.alvarsson@karolinska.se  Marie Degerblad, MD.  Dept. of Endocrinology, Metabolism and Diabetes, Karolinska University Hospital  Phone: +46-8-517 700 00  E-mail: marie.degerblad@karolinska.se | **Scientific Advisor**  Suad Efendic, Prof. emeritus,  Dept. of Molecular Medicine and Surgery  Karolinska Institutet  171 76 Stockholm, Sweden  Phone: +46-8-517 743 65  E-mail: suad.efendic@ki.se    **Research nurses**  Kajsa Sundqvist, Maria Wärn, Mirjam Rhyner  Enheten för Metabol kontroll, Dept. of Endocrinology, Metabolism and Diabetes  Karolinska University Hospital  171 76 Stockholm, Sweden  Phone: +46-8-517 725 35  Fax: +46-8-517 739 68  E-mail: kajsa.sundqvist@karolinska.se  **Monitor** Monitour AB  Smedjegatan 7  652 18, Karlstad, Sweden  Lennart Jönsson  Phone: +46-54-21 75 75  Mobile: +46-70-742 65 63  E-mail: info@monitour.se |
| --- | --- |

1. Background Information
   1. Background and Rational

Diabetes mellitus is a heterogeneous disease and about 90 percent of patients may be estimated to have type 2 diabetes (T2D) [1]. The disease is associated with development of severe microvascular and macrovascular complications [2] . Hence, diabetes may account for approximately 10-15 percent of total health care costs in high-income countries [3]. There is an increased incidence of T2D, in particular in low-income and developing countries, which might be due to introduction of new, modern lifestyle in combination with genetic background [1, 4]. To meet a global epidemic of T2D, major efforts are accomplished to practice known and find novel approaches for prediction and prevention of the disease. Thus, it is well established that enhanced physical activity and modest weight reduction can significantly prevent or delay development of manifest diabetes in subjects with impaired glucose tolerance, IGT [5].

Glucose intolerance develops on the basis of impaired insulin secretion and decreased insulin sensitivity, due to interaction between environmental and genetic factors [4, 6]. Importantly, recent genome-wide association studies have shown that the genetic predisposition to T2D is mainly linked to genes associated with B-cell function, while only a few genes may be regulating insulin sensitivity [7]. Most lifestyle factors, such as obesity, physical inactivity, psychosocial stress and tobacco use, seem to be able to decrease insulin sensitivity [4, 8-12]. In addition, it is possible that impairment of insulin secretion is further accounted for by some of these factors, e.g. tobacco and stress. When lifestyle changes induce decrease in insulin sensitivity in a person with T2D heredity, hyperglycaemia may occur since the β-cells cannot compensate for the insulin resistance by increasing insulin secretion.

In addition to the above well-documented diabetogenic factors, there is a growing body of evidence that soluble inflammatory factors, including cytokines and chemokines, may be associated with increased risk of developing T2D [13]. Several of these factors also appear to be predictors of insulin resistance and cardiovascular diseases. Furthermore, there is accumulating evidence suggesting that altered vitamin D and calcium homeostasis may play a pivotal role in the pathogenesis of T2D. A recent meta-analysis of the role of vitamin D and calcium in T2D has concluded that observational studies demonstrate a relatively consistent association between low vitamin D serum levels and prevalence of T2D or metabolic syndrome [14]. Moreover, a Finnish cohort study showed an inverse association between baseline serum 25-hydroxy-vitamin D3 (25(OH)D) and 17-year risk of type 2 diabetes, which was attenuated after adjusting for confounders [15]. Similarly, the Ely Prospective Study demonstrated inverse associations in 524 non-diabetic men and women, aged 40-69 years at baseline, between serum 25(OH)D levels at baseline and measures of glycaemia and insulin resistance ten years later [16].

As to intervention studies, there are limitations due to small sample size and short intervention periods. There are only three interventional studies with more than 100 participants treated with vitamin D during 2-3 years [17-19] . In one study, vitamin D3 at a daily dose of 2000 IU had no effect in a subgroup of 25 subjects [17]. In a post hoc analysis of a trial focusing on bone-related parameters, vitamin D therapy with a daily dose of 700 IU improved insulin sensitivity measured with HOMA in IGT subjects [18]. Finally, in a Women´s Health Initiative study a low daily vitamin D dose of 400 IU had no effect on the risk of developing T2D [19]. The latter study, however, not only used a low dose of vitamin D, but results were not adjusted for control subjects´ intake of vitamin D. In summary, the intervention trials have had short duration, in rather few subjects, used various types and doses of vitamin D and calcium, or did post hoc analyses.

Several human studies have targeted the question if intervention with vitamin D could affect beta cell function and/or insulin sensitivity. As to beta cell function, some studies have shown beneficial effects [20-23], whereas some have not [24-27]. These studies are in many cases limited by a non-randomized or placebo controlled design, small samples, intervention with 1,25-dihydroxy-vitamin D (1,25(OH)_2_D) (which theoretically may be less favorable than 25(OH)D) and indirect measures of insulin secretion. Most of these studies used oral glucose tolerance test (OGTT) or HOMA-β as assessment instead of hyperglycemic clamp, which is considered as the golden standard. Two studies used the more sophisticated intra-venous glucose tolerance test (IVGTT). With this method, Borrissova et al. found an increased first-phase insulin secretion after 1 month treatment with 1332 IU cholecalciferol/day in 10 women with T2D, but the study was not placebo-controlled [22]. The likewise not placebo-controlled 4 days short study by Zofkova et al. with 3 µg 1,25(OH)_2_D/day in 13 healthy adults showed no change in insulin secretion [24]. As to insulin sensitivity, the reported studies have also shown conflicting results. A few studies have shown an improvement in insulin sensitivity [18, 27], but several could not [19, 22, 26, 28-31]. Studies using fasting plasma values to indirect assess insulin sensitivity have in general been negative, whereas studies using OGTT often have been limited by small sample size and short intervention period. The three studies using more sophisticated methods for assessment of insulin sensitivity (IVGTT or euglycemic clamp) [29-31] showed no effect but used 1,25(OH)_2_D or active analogues as supplementation.

To summarize, there is conflicting evidence of the potential role of vitamin D in this field and therefore a need for randomized, placebo controlled trials with sufficient sample size and gold standard methods for the assessments.

Results of experimental and clinical studies suggest that plasma levels of insulin-like growth factor-1 (IGF-1), and insulin-like growth factor binding protein-1 (IGFBP-1) could be important determinants of glucose homeostasis [32, 33]. Recently, we have demonstrated, in the Stockholm Diabetes Prevention Programme (SDPP), that low serum IGFBP-1 levels in men with normal glucose tolerance at baseline strongly predicted risk of developing T2D and prediabetes at a follow-up study 10 years later [33]. In the study of Hypponen et al [34], there was evidence of interaction between 25(OH)D and IGF-1 plasma levels, suggesting that metabolic syndrome prevalence is the lowest when both 25(OH)D and IGF-1 levels are high. In contrast, the recent 10-year prospective study of Forouhi and coworkers [16] could not confirm this interaction between IGF-1 and vitamin D. The latter study, however, showed that high serum 25(OH)D concentrations attenuated fasting and 2h-glucose levels of OGTT in subjects with low, but not high, IGFBP-1 levels. Thus, these data suggest that biological interactions between the IGF-1 and/or its binding proteins on one hand and vitamin D on the other are important in the regulation of glucose homeostasis.

As to the possible mechanisms behind effects of vitamin D on glucose homeostasis, vitamin D receptors are expressed in more than 30 different tissues, including pancreatic islet cells [35]. Vitamin D deficiency suppresses pancreatic secretion of insulin [36], suggesting that vitamin D treatment may improve glucose tolerance by restoring impaired insulin release present in subjects with impaired glucose tolerance and manifest T2D. It is unclear whether this effect is mediated by direct action of vitamin D or through modulation of calcium metabolism. Further, the 1-α hydroxylase enzyme is present in β-cells, which converts 25(OH)D to the active 1,25(OH)_2_D [37]. This may implicate that supplementation with active 1,25(OH)_2_D is inferior to vitamin D_2_ or D_3_, which serve as substrate for the enzyme.

Animal and human studies suggest that vitamin D treatment may improve insulin sensitivity. Vitamin D receptors are also present in skeletal muscle [38], stimulate the expression of insulin receptor and enhance insulin responsiveness for glucose transport in vitro [39]. Furthermore, there is some evidence that polymorphisms in the vitamin D receptor gene are linked to insulin resistance, insulin secretion, and fasting plasma glucose levels [40-43].

- 1. Preliminary data

Within the Stockholm Diabetes Prevention Programme (SDPP), a baseline study was performed during 1992-1998 . The prevalence of diagnosed T2D and family history of diabetes was mapped by a questionnaire to all subjects, 35-55 years old, in five Stockholm County municipalities. From this sample, a total of 3128 men (1992-1994) and 4821 women (1996-1998), none with previously diagnosed diabetes, but 50% with family history of diabetes (FHD), were investigated with oral glucose tolerance test (OGTT), body measurements (weight, height, waist, blood pressure etc), and an extensive questionnaire about lifestyle (i.e. food, exercise, tobacco, alcohol habit) and psychosocial and socio-economical issues related to working and family life, education, social network, and stress. In this baseline study, we found 65 men with diabetes (2.1%) and 228 men with prediabetes (IFG and/or IGT) (7.3%), and 63 women with diabetes (1.3%) and 208 with prediabetes (4.3%).

In the follow-up study 8-10 years later (2003-2006), we repeated a similar investigation, including OGTT and the same questionnaire as at baseline, in more than 70% of the baseline study participants. At follow-up, like at baseline, men had a two-fold higher prevalence of T2D and prediabetes (7.7% and 12.0%, respectively) compared with women (3.3% and 6.2%, respectively). Thus, of men with normal glucose tolerance at baseline we found a total of 111 with diabetes and 255 with prediabetes, and of women with normal glucose tolerance at baseline we found 60 with diabetes and 181 with prediabetes. In addition, there were 72 men and 46 women with prediabetes at baseline, who had developed manifest T2D at follow-up.

1. Endpoints
   1. Primary Endpoint

To test whether there is a difference in the mean change in first-phase plasma insulin response at the hyperglycemic clamp investigation [44] at visit 4 (8 weeks) compared to baseline, between patients receiving vitamin D3 30000 IU weekly for 8 weeks vs. placebo. Insulin will be measured at minutes 0, 2, 4, 6, 8 and 10. Increment from minute 0 in area-under-curve (AUC) will be computed and used for the statistical analyses.

- 1. Secondary Endpoints
     1. Secondary Efficacy Endpoints

To test whether there is a difference in the mean change in late phase insulin response (10-120 minutes) during hyperglycemic clamp at visit 4 (8 weeks) compared to baseline, between patients receiving vitamin D3 30000 IU weekly for 8 weeks vs. placebo.

To test whether there is a difference in the mean change in insulin sensitivity assessed as the ratio of the glucose infusion rate and plasma insulin concentration (M/I) during hyperglycemic clamp at visit 4 (8 weeks) compared to baseline, between patients receiving vitamin D3 30000 IU weekly for 8 weeks vs. placebo.

To test whether there is a difference in the mean change in glucose tolerance assessed by an oral glucose tolerance test (OGTT) at visit 4 (8 weeks) compared to baseline, between patients receiving vitamin D3 30000 IU weekly for 8 weeks vs. placebo.

To test whether there is a difference in the mean change in fasting plasma glucose and HbA1c at visit 4 (8 weeks) compared to baseline, between patients receiving vitamin D3 30000 IU weekly for 8 weeks vs. placebo.

To test whether there is a difference in the mean change in CVD risk markers/lipids at visit 4 (8 weeks) compared to baseline, between patients receiving vitamin D3 30000 IU weekly for 8 weeks vs. placebo.

To test whether there is a difference in the mean change in hormones as leptin, adiponectin and GLP-1 at visit 4 (8 weeks) compared to baseline, between patients receiving vitamin D3 30000 IU weekly for 8 weeks vs. placebo.

To test whether there is a difference in the mean change in 25-OH-vitamin D and PTH at visit 4 (8 weeks) compared to baseline, between patients receiving vitamin D3 30000 IU weekly for 8 weeks vs. placebo.

- - 1. Safety Endpoints

To compare the intervention with vitamin D3 compared to placebo in:

- Incidence of hypercalcemia defined as free s-calcium > 1.35 mmol/l at week 4 and 8.
- Laboratory assessments (hematology, biochemistry).
- Physical examination.
- Adverse events regarding incidences, intensity, severities and causality.

1. Design
   1. Outline

The trial is a randomized, parallel groups, double-blind, placebo-controlled trial with an 8 weeks intervention period. The trial will include a screening visit to assess the eligibility of the subjects. At the randomization visit the baseline investigations will be performed and the subjects will be randomized to one of two intervention arms:

- In the first treatment arm (arm A), subjects will receive vitamin D3 intervention, 30000 IU given orally once weekly. After 8 weeks the investigations will be repeated.
- In the second treatment arm (arm B), subjects will receive matching placebo once weekly. After 8 weeks the investigations will be repeated.

Figure: Trial design

- 1. Visits and telephone contacts
- Visit 1 (Screening, week -2): Patient information, informed consent and eligibility. OGTT.
- Telephone 1 (week -1): Eligibility.
- Visit 2 (Randomization, week 0): Hyperglycemic clamp. First dose of study treatment.
- Telephone 2 (week 1). Compliance. Second dose of study treatment.
- Telephone 3 (week 2). Compliance. Third dose of study treatment.
- Telephone 4 (week 3). Compliance. Fourth dose of study treatment.
- Visit 3 (week 4). Safety and fifth dose of study treatment.
- Telephone 5 (week 5). Compliance. Sixth dose of study treatment.
- Telephone 6 (week 6). Compliance. Seventh dose of study treatment.
- Telephone 7 (week 7). Compliance. Eighth dose of study treatment.
- Visit 4 (week 8+0d and week 8+1d). End-of-study investigations (OGTT + hyperglycemic clamp).
- Telephone 8 (week 9). Follow-up safety.
  1. Assessments and Procedures
     1. Recruitment of subjects

Subjects are recruited from the database of the SDPP. Subjects who at follow-up were categorized as having IFG, IGT or IFG + IGT (N=366) at the follow up performed in the years 2003 - 2006 will be contacted for the present study. The screening period will start in Q3 2011. An OGTT will be performed at screening and subjects still having one of the three pre-diabetic conditions, or diet treated diabetes type 2 will be eligible for the study.

1. Selection and Withdrawal of Subjects

The sample size estimation (see section 12.3) requires that 38 subjects should complete the study to investigate the primary endpoint. To compensate for study drop-out, a total of 44 male or female subjects will be included in the study. With a screening failure rate of 30 %, about 58 subjects need to be screened.

- 1. Inclusion Criteria

1. Informed consent obtained before any trial-related activities[[1]](#footnote-2).
2. Meeting criteria for IFG, IGT, IFG+IGT or diabetes mellitus at the screening OGTT.
   - IFG = fasting p-glucose 6.1-6.9 mmol/l
   - IGT = 2 hour p-glucose 7.8-11.0 mmol/l
   - Diabetes = fasting p-glucose ≥ 7.0 mmol/l and/or 2 hour p-glucose ≥ 11.1 mmol/l
3. Age ≥ 45 and ≤ 75 years, female or male.
4. BMI ≤ 32 kg/m^2^.
5. HbA_1c_  ≤ 7.0 % (MonoS) or ≤ 63 mmol/mol (IFCC).
6. Fasting plasma glucose < 9 mmol/l.
7. S-25-OH-vitamin D3 < 75 nmol/l.
8. Able and willing to perform tests and examinations specified in the protocol.
   1. Exclusion Criteria
9. Previous participation in this trial. Participation is defined as randomization.
10. Anticipated change in dose of concomitant medication which may interfere with glucose metabolism, such as systemic corticosteroids, non-selective beta-blockers, mono amine oxidase (MAO) inhibitors and anabolic steroids.
11. Treatment with any vitamin D preparation.
12. Regular sun-bathing in solarium.
13. Hypercalcemia at screening, defined as free s-calcium > 1.35 mmol/l.
14. Hyperphosphatemia at screening, defined as s-phospate > 1.5 mmol/l.
15. Sarcoidosis or other granulomatous disease.
16. All contraindications to vitamin D treatment.
17. Treatment with phenytoin, barbiturates, rifampicin, isoniazid, cardiac glycosides, orlistat or colestyramin.
18. Impaired hepatic function defined as alanine aminotransferase (ALAT) ≥ three times the upper reference limit.
19. Impaired renal function defined as S-creatinine >133 µmol/L for males and >115 µmol/L for females.
20. Cardiac disease defined as:
    1. Unstable angina pectoris
    2. Myocardial infarction within the last 6 months
    3. Congestive heart failure NYHA class III and IV
21. Cerebral stroke within the last 6 months.
22. Uncontrolled treated/untreated hypertension (systolic blood pressure ≥ 180 mmHg and/or diastolic blood pressure ≥ 110mmHg).
23. Cancer (except basal cell skin cancer or squamous cell skin cancer).
24. Anti-diabetic medication of any kind.
25. Females of childbearing potential who are pregnant, breast-feeding or intend to become pregnant or are not using adequate contraceptive methods. Menopause is defined as > 1 year since last menstruation..
26. Known or suspected abuse of alcohol or narcotics.
27. Mental incapacity, unwillingness or language barrier precluding adequate understanding or co-operation.
28. Any other condition that the Investigator and/or Sponsor feel would interfere with trial participation or evaluation of results.

At patient inclusion, the text “inklusionskriteria och exklusionskritera har kontrollerats och funnits uppfyllda” will be documented in the patient records instead of listing every separate item.

- 1. Criteria for Withdrawal

The subject may withdraw at will at any time during the study without declaring any reason.

The subject may be withdrawn from the trial at the discretion of the Investigator if judged non-compliant with trial procedures or due to a safety concern.

A subject must be withdrawn if the following applies:

1. Occurrence of hypercalcemia during the study, defined as free s-calcium > 1.35 mmol/l. In case of hypercalcemia, a second test should be performed a soon as possible to verify the diagnosis.
2. Initiation of concomitant medication known to interfere with glucose metabolism, such as systemic corticosteroids, non-selective beta-blockers, mono amine oxidase (MAO) inhibitors and anabolic steroids.
3. Initiation of any anti-diabetic medication.
4. Initiation of treatment with phenytoin, barbiturates, rifampicin, isoniazid, cardiac glycosides, orlistat or colestyramin.
5. Pregnancy or intention to become pregnant in female subjects.
   1. Subject Log

All screened subjects will be documented. For subjects who either fail screening or are withdrawn prior to randomization, a Screening Failure Form must be completed including the reason for screening failure.

1. Treatment
   1. Description of Investigational Medicinal Products
      1. Vitamin D3 (Cholecalciferol), Vigantol® Oil

Vitamin D3 will be supplied to the study site in bottles of 10 ml (200 000 IE). Vitamin D3 will be provided by Renapharma AB.

**Dosage form**: Mixture. 20 000 IU/ml (0.5 mg/ml)

**Administration**: Oral.

**Dose**: 30000 IU (1.5 ml) once weekly (corresponding to 4286 IU daily) for 8 weeks.

**Manufacturer**: Merck KGaA

- - 1. Placebo

Matching placebo (oil) will be supplied to the study site in bottles of 10 ml. Placebo will be provided by Renapharma AB.

**Dosage form**: Mixture.

**Administration**: Oral.

**Dose**: 1.5 ml once weekly, for 8 weeks.

**Manufacturer**: Merck KGaA

- 1. Packaging, Labeling, Storage and Handling of Investigational Medicinal Products

The IMP will be supplied to the Karolinska pharmacy who will conduct, in co-operation with Apotek Produktion & Laboratorier AB (APL), the labeling and storage. The IMP will be provided to the study site at study start when all approvals have been granted. The labeling will contain information on the name of the study, protocol number, EudraCT-number, dosing, IMP information (name, strength, form, route of administration and amount), batch number, randomization number and initials, keeping instruction and expiration date, warning text that keep away from children, primary investigator, sponsor and the text “För klinisk prövning”. Also see amendment C.

- 1. Treatment Assignment

The randomization will take place at visit 2 (randomization visit) just prior to initiation of trial treatment. Randomization will be carried out in a 1:1 manner and administered by the Investigator. The treatment assignment will be double-blind. A randomization list will be prepared by a statistician not involved in the study. The list will contain randomization numbers 1 to 44 and corresponding study medication (active substance or placebo) The study medication will be marked with a randomization number, 1 to 44, by APL according to the randomization list. Subjects that are included in the study will sequentially be assigned a randomization number, and thereby the corresponding study medication. Unblinding can be carried out in medical emergencies by the Investigator. Individual treatment codes will be available to the Investigator by personnel at the department not involved in the study. A list with the treatment codes will be kept at Enheten för Metabol kontroll, Endokrinliniken D1:03, Karolinska Universitetssjukhuset Solna, phone +46-8-517 725 35.

- 1. Concomitant Medication

Details of all concomitant medication must be recorded at the screening visit. This should include the start date of medication, and name Subjects will record any changes in concomitant medication in the diary and the investigator will transcribe the information into the CRF at all visits.

- 1. Compliance to Treatment

The IMP will be given by a study nurse at visits to the study site at study weeks 0 and 4. The subjects will take IMP at home at weeks 1,2,3,5,6 and 7. Telephone contacts will be carried out at these time points to optimize compliance. If study subject prefer SMS reminder, this could be done instead of a phone call. The SMS should be confirmed by the patient. Subjects will bring all IMP bottles to the study site for compliance assessment at visit 3 and 4. Serum 25-OH-vitamin D will also be analyzed at week 4 and 8.

- 1. Continuation of Treatment

There will be no active post-treatment follow-up period. At the last visit the subjects will receive information and counseling regarding treatment of their glycemic disturbance. Information will be sent to the subjects´ physicians.

1. Assessments and procedures
   1. Efficacy Assessments

All efficacy assessments will be carried out before and at the end of the intervention period if not stated otherwise.

- - 1. Hyperglycemic clamp
       The plasma insulin response (I) during hyperglycemic clamp will assess beta cell function [44]. Glucose infusion is administered at a variable rate to increase the plasma glucose to a target value 6.9 mmol/l above the fasting glucose value. Blood samples for glucose and insulin are taken every 2 minutes from minute 0 to 14 and every 5-10 minutes from minute 10 to 120. The ratio of the glucose infusion rate and plasma insulin concentration (M/I) will assess insulin sensitivity. Further, extra archive serum samples will be collected at 0, 10, 60 and 120 minutes.
    2. Oral glucose tolerance test (OGTT)

A sample for fasting plasma glucose and serum insulin will be obtained (0 minutes). 75g of glucose in water will then be given orally. Plasma glucose and serum insulin will be obtained at 30 and 120 minutes after the glucose administration. Glucose will be analyzed in local laboratory by a glucose oxidase method. Further, extra archive serum samples will be collected at 0, 30 and 120 minutes.

- - 1. HbA1c

HbA1c will be analyzed using the high-performance liquid chromatography Mono-S method at Karolinska central laboratory.

- - 1. CVD riskmarkers and Lipids

Serum triglycerides, total cholesterol, HDL, Apo B and Apo A1 will be obtained in fasting state and analyzed at Karolinska central laboratory.

- - 1. Hormones

Serum GLP-1, adiponectin and leptin will be obtained in fasting state and analyzed in local laboratory.

- - 1. 25-OH-vitamin D and PTH

Analyses will be performed at Karolinska central laboratory . 25-OH-vitamin D will also be assessed at week 4 (visit 3).

- - 1. Body composition

Weight, length, BMI and bioimpedance (by Tanita-Body Composition Analyzer).

- - 1. Archive samples

Fasting plasma samples and end-clamp samples for future research will be obtained and frozen at visit 2 and 4.

- 1. Safety Assessments

Adverse events will be recorded at every visit/contact, except for the screening visit. All other safety assessments will be carried out before and at the end of the intervention period if not stated otherwise.

- - 1. Physical Examination

A general physical examination will be carried out at screening and at study end, including: cardiovascular system, respiratory system, gastrointestinal system, central and peripheral nervous system, musculoskeletal system and skin, according to common procedure. Any clinically significant deterioration since the screening visit should be regarded as an adverse event.

- - 1. Hematology and Biochemistry

This include hemoglobin, leukocytes, thrombocytes, sodium, potassium, phosphate, creatinine, albumin, ALAT and ALP.

- - 1. Free Calcium

Free s-calcium will also be assessed at week 4 (visit 3).

- - 1. Pregnancy test

This will be carried out in female subjects with child bearing potential.

- - 1. Vital signs

Pulse and blood pressure will be measured at visits 1, 2, 3 and 4.

- - 1. Concomitant Illness and Medication (including current diabetes treatment)

Details of all concomitant illnesses and medication must be recorded at trial entry (screening visit).

Subjects will record any changes in concomitant medication in the diary and the investigator will transcribe the information into the CRF at all visits.

- 1. Other assessments
     1. Life Style

All subjects will be instructed to maintain their diet and their level of physical activity during the study.

- - 1. Step count

Subjects will be equipped with a step-count device. They will be instructed to record step-count for 7 days before visit 2 and visit 4.

- - 1. Body composition

Height will be measured at visit 1. Weight will be measured at visit 1, 2 and 4. Bioimpedance by Tanita Body Composition Analyzer will be measured at visit 2 and 4. Waist will be measured at visit 2 and 4.

- - 1. Information of results

All subjects will receive a letter after the trial, presenting a subset of their personal results.

See also Amendment B.

1. Proceedings for Adverse Events
   1. Definition of Adverse Events
      1. Definition of Adverse Events

An Adverse Event (AE) is any untoward medical occurrence in a subject administered Investigational Medicinal Products (IMP) and which does not necessarily have a causal relationship with this product. An AE can be any unfavorable and unintended sign, abnormal laboratory finding, symptom or disease temporally associated with the use of IMP, whether or not related to the product.

- - 1. Definition of Adverse Reactions

Each AE is to be classified by the investigator as related or not related to the IMP. An Adverse Reaction (AR) is a noxious and unintended medical ***response*** to a medical product related to any dose. For an AE to be an AR the suspected association between the product and the unwanted medical condition should be at least a reasonable ***possibility***.

- - 1. Definition of Serious Adverse Events

Each AE is to be classified by the investigator as serious or non-serious. Seriousness is not defined by a medical term; it is a result or an outcome. An AE is defined as a Serious Adverse Event (SAE) if it:

- results in death
- is life-threatening
- requires inpatient hospitalization or prolongation of existing hospitalization
- results in persistent or significant disability/incapacity
- results in a congenital anomaly/birth defect
- important medical events that may not result in death, be life-threatening, or require hospitalization may be considered an SAE when, based upon appropriate medical judgment, they may jeopardize the subject and may require medical or surgical intervention to prevent one of the outcomes in this definition.
  - 1. Definition of Serious Adverse Reactions

This is defined as an adverse reaction (AR) fulfilling any seriosity criterion as for SAE (see 11.1.3).

- - 1. Definition of Suspected Unexpected Serious Adverse Reactions

Each serious adverse reaction (SAR) that is at least possibly related to IMP is to be classified by the investigator as expected or unexpected. A SAR that is at least possibly related to IMP, and ***unexpected***, is defined as a Suspected Unexpected Serious Adverse Reaction (SUSAR). It is expected if it is already known from earlier trials or is mentioned in relevant documents (SPC´s).

- 1. Assessment of Adverse Events
     1. Assessment of Intensity

Each AE is to be classified by the investigator as mild, moderate or severe.

**Mild:** Acceptable. The subject is aware of symptoms or signs, but they are easy tolerated.

**Moderate:** Disturbing. The AE is discomforting enough to interfere with usual daily activity.

**Severe:** Unacceptable. The subject is incapacitated to work or to do usual daily activities.

- - 1. Assessment of Causality

**Unlikely:** The event is most likely related to an etiology other than the IMP.

**Possible:** A causal relationship is conceivable and cannot be dismissed.

**Probably:** Good reason and sufficient documentation to assume a causal relationship.

- - 1. Assessment of Pregnancy

Pregnancy occurring during the trial will not be considered as a SAE. If a trial subject becomes pregnant during the trial period (the screening period excluded), the investigator must report information on pregnancy, pregnancy outcome and health status of the infant to the MPA. The subject will be excluded from the study.

- 1. Methods for Eliciting Adverse Events

All events meeting the definition of an adverse event must be collected and reported from the first trial related activity after the subject has signed the informed consent and received the first dose of IMP, and until the end of the study. At each contact with the site (visit or telephone), the subject must be asked about adverse events. All adverse events, either observed by the Investigator or reported by the subject, must be recorded by the Investigator and evaluated.

The subject will be asked about adverse events, with a question: “Have you experienced any problems since the last contact? (Swedish: Har du upplevt några besvär sedan vår senaste kontakt?)”

The Investigator should record the diagnosis, if available. If no diagnosis is available the Investigator should record each sign and symptom as individual adverse events.

All adverse events must be recorded by the Investigator on the standard Adverse Event Form. If more than one sign or symptom is to be reported, use a separate Adverse Event Form for each sign and symptom. For serious adverse events, the Serious Adverse Event Supplementary pages must also be completed.

- 1. Reporting of Adverse Events
     1. Reporting of Adverse Events

All AEs will be recorded on a separate AE form in the CRF.

- - 1. Reporting of Serious Adverse Events

In addition will SAEs also be reported by the investigator to the sponsor on a separate SAE form within 24 hours after the SAE have been communicated to the investigator. Follow-up information describing the outcome of the SAE and action taken will be reported as soon as it is available. The original SAE form must be filed with the CRF. The sponsor shall report all cases of death to the MPA within 7 days, and if necessary complete the report to the MPA within 8 days and all cases of death to the Independent Ethics Committee (IEC).

- - 1. Reporting of Suspected Unexpected Serious Adverse reactions

The sponsor must report all SUSARs that resulted in death or was life threatening to the authority through the EudraVigilance database and to the IEC within 7 days, and if necessary complete the report within the following 8 days. Other SUSAR should be reported within 15 days. A CIOMS form will be sent to the MPA, which in turn will report to the EudraVigilance database.

- 1. Follow-up of Adverse Events

During and following a subject’s participation in the trial, the Investigator should ensure that adequate medical care is provided to the subject for any adverse events, including clinically significant laboratory values related to the trial. The Investigator should inform the subject when medical care is needed for adverse event(s) of which the Investigator becomes aware.

The follow up information should only include new (updated and/or additional) information that reflects the situation at the time of the investigator’s signature.

All AEs, SAEs and SUSARS will be followed until they are classified as “recovered” or “stable” and/or in the care of medical expertise.

1. Statistics and Data Management
   1. Data Management

The subjects and the biological material obtained from the subject will be identified by initials, screening/randomization number, trial site, and trial identification number. Appropriate measures such as encryption or deletion will be enforced to protect the identity of human subjects in all presentations and publications as required by local/regional/national requirements.

Data from CRFs and other source documents will be transferred manually to a database by Henrik Wagner. A subset of the transferred data will be controlled against source data for verification. Initials and randomization number will be used for identification in the database.

- 1. Statistical Analysis

The Intention-To-Treat (ITT) population will be included in the safety analysis. The ITT population will consist of all randomized subjects who receive at least one dose of IMP. An analysis based on the Per-Protocol (PP) population will be performed on the efficacy variables. The PP population will consist of subjects exposed to trial drug who did not significantly violate the inclusion/exclusion criteria or other aspects of the protocol considered to potentially affect the primary endpoint. Data from drop-outs, withdrawals and non-compliant subjects will hence not be included in the PP analysis.

It is the joint responsibility of the Principal Investigator and the Scientific Advisors to decide on exclusion of observations or subjects from the analysis.

The subjects or observations to be excluded, and the reasons for their exclusion must be documented and signed by the above mentioned persons prior to closing the database. The documentation must be stored together with the remaining trial documentation.

Unless otherwise specified, all tests will be two-sided at 5 % significance level. Correction for multiple secondary endpoints will not be performed. Results of all endpoints will be presented irrespective of statistical significance. Further, all primary and secondary endpoints will also be summarized by descriptive statistics.

- - 1. Endpoints

See section 6.

- - 1. Analysis of efficacy endpoints

The primary efficacy analysis will test for differences between arm A and B in the change from baseline in insulin response at the hyperglycemic clamp investigation after 8 weeks of treatment. The non-parametric Mann-Whitney U test will be used primarily. The Wilcoxon matched pairs test will be used for within group analyses. If data permits (i.e. normal distribution), parametric tests will also be used. Multiple regression analysis will be performed to adjust for age, sex and other baseline characteristics, if necessary.

- - 1. Analysis of safety endpoints

#### Adverse events, serious adverse events and adverse reactions

Adverse reactions and serious adverse events will be coded using Medical Dictionary for Regulatory Activities (MedDRA) and summarized by treatment group, MedDRA system organ class, MedDRA preferred term, severity and relation to trial product. All adverse events will be listed.

For an AE to be an AR the suspected association between the product and the unwanted medical condition should be at least a reasonable ***possibility***. This include if they occur between first trial drug date and until 7 days after last trial drug date. Events occurring outside this interval are not classified as AR; hence they will not be included in the summaries.

#### Other safety endpoints

Other safety endpoints will summarized by descriptive statistics:

- Clinically relevant change in physical examination
- Occurrence of hypercalcemia, defined as free s-calcium > 1.35 mmol/l
- Occurrence of plasma levels of 25-OH-Vitamin D > 250 nmol/l
- Clinically relevant change in plasma hematology
- Clinically relevant change in plasma biochemistry
  1. Determination of Sample Size

The present study is considered as a pilot study. No similar publicized study has been found that could provide data for a formal power calculation. A recent randomized trial with a cytokine suppressor could demonstrate a significant increase first phase insulin secretion, the effect being slightly less than 30 % [45]. In this study, treatment and control groups comprised of 20 individuals each. Several previous studies performed at our own unit on insulin sectretion and insulin sensitivity have been successfully carried out in groups of 10-15 individuals. We will therefore expect that18 subjects should be available for analysis in each arm. To allow for using a non-parametric test about 5 % more patients are needed (Lehmann 1975) and thus 19 subjects in each arm should be included in the final analysis. To compensate for study drop-out, 22 subjects should be randomized to each treatment arm. Thus 44 subjects should be included in the study. With a screening failure rate of 30 %, about 58 subjects need to be screened.

1. Direct Access to Source Documents

The Investigator has the responsibility that a secrecy agreement is established between the clinic and the monitor, giving the permission to verify data in the patients´ hospital records. Signed informed consent will be obtained from the subjects prior to any trial-related activity regarding this matter.

1. Quality Control
   1. Source Data

Information in each patients hospital records will include information on study participation study title, randomization number, informed consent, diagnose, the two different study treatments, who to contact regarding questions about the trial and where the randomization codes are kept.

- - 1. The following template will be used at visit 1:

“Patienten kommer idag för screeningbesök i studien ”***Effects of vitamin D on beta cell function and insulin sensitivity in pre-diabetes and diabetes mellitus type 2 – EVIDENS”,*** en dubbelblind placebokontrollerad studie för att studera effekt av oralt vitamin D3 30000 IU eller placebo, oralt en gång per vecka i 8 veckor, på betacellsfunktion samt insulinkänslighet. Patienten får idag information om studien och lämnar skriftligt informerat samtycke till deltagande. Information kring studien kan inhämtas från ansvarig prövare Henrik Wagner, Endokrinkliniken, Karolinska Solna, 08-517 793 38 alternativt forskningssköterska, Enheten för Metabol kontroll, Endokrinkliniken, Karolinska Solna, 08-517 725 35. Vid Enheten för Metabol kontroll förvaras randomiseringslista vid akut behov att bryta randomiseringskod.

Patienten får screening-nr:

Inklusionskriterier och exklusionskriterier har kontrollerats och hittills funnits uppfyllda. Svar på lab-prover inväntas.”

- - 1. The following template will be used at telephone contact 1:

“Telefonkontakt 1 i EVIDENS. Inklusionskriterier och exklusionskriterier har kontrollerats och funnits uppfyllda. Patienten planeras in för visit 2 (randomiseringsbesök)”

- - 1. The following template will be used at visit 2:

“Patienten kommer idag för visit 2 (randomiseringsbesök) i EVIDENS. Patienten randomiseras och erhåller randomiserings-nr: ”

- - 1. For visits 3 and 4, the following information should be stated:
- State visit number and possible adverse events.
- State if the patient has completed the study, or discontinued prematurely.
- In case of premature discontinuation of the trial, ask for the reason, enter the information in the patient’s medical file if the patient accepts to disclose this information.
- After the study: The nature of the treatment given, active or placebo, has to be written in the patient records and this information is to be released to the patient when all patients have completed the study and all data have been checked.
  1. Monitoring

During the course of the study, monitoring will be performed in accordance with the Good Clinical Practice (GCP) guidelines and Swedish regulations. Study conductance, source data and adherence to regulatory requirements and protocol will be monitored. A study initiation visit will be performed before start of the study.

1. Ethics
   1. Independent Ethics Committee

The Investigator is obligated to submit to the local Independent Ethics Committee (IEC), prior to commencement of the trial:

- The protocol and any amendments concerning Informed consent, Subject information and the suitability of the Investigator.
- Subject Information/Informed Consent Form and any other written information to be provided to the subject.
- Subject recruitment procedures.
- Information about payments and compensation available to subjects if not mentioned in the subject information.
- The Investigator’s current CV and/or other documentation evidencing qualifications.
- Other documents as required by the local IEC.

Written approval/favorable opinion must be obtained from IEC prior to commencement of the trial. The Sponsor is responsible for sending obtained approval from the IEC to the Medical Product Agency (MPA).

During the trial, the Investigator must promptly report the following to the IEC:

- Unexpected SAEs where a causal relationship cannot be ruled out.
- Substantial amendments to the protocol.,
- Deviations to the protocol implemented to eliminate immediate hazards to the subjects.
- New information that may affect adversely the safety of the subjects or the conduct of the trial (including new risk/benefit analysis in case it will have an impact on the planned follow-up of the subjects).
- Annually written summaries of the trial status and other documents as required by the local IEC.

Substantial amendments must not be implemented before approval/favorable opinion, unless necessary to eliminate hazards to the subjects. The Investigator must maintain an accurate and complete record of all submissions made to the IEC.

- 1. Ethical Conduct of the Trial

The Investigator must comply with the applicable regulatory requirement(s) and adhere to the ICH GCP guideline and the requirements in the Declaration of Helsinki.

- 1. Subject Information and Informed Consent

Prior to any trial-related activity, the Investigator must give the subject oral and written information about the trial in a form that the subject can read and understand.

A voluntary, personally signed and dated Informed Consent Form will be obtained from the subject prior to any trial-related activity by the Investigator.

The written informed consent must be signed and dated by the person who conducted the informed consent procedure.

If information becomes available that may be relevant to the subject’s willingness to continue participating in the trial, the Investigator must inform the subject in a timely manner, and a revised written informed consent must be obtained.

The Sponsor must ensure that the signed Informed Consent Form is obtained from the subjects.

- 1. Risk assessment

In a review published in 2007 of available studies, no toxic effects were seen in healthy human subjects receiving a dose of vitamin D3 corresponding to ≥ 10000 IU daily [46]. Eight weeks of daily intake of 10000 IU resulted in a mean serum 25-hydroxyvitamin D (25(OH)D) level of 213 nmol/l. This is well below the potential toxic level of > 700 nmol. In studies using doses comparable to the present study (4000 IU daily), the highest serum level of 25(OH)D was 126 nmol/l. Seven identified studies published after 2007 supports these findings [47-53]. In one cancer study, the treatment with vitamin D unmasked two cases of primary hyperparathyroidism, but no toxic effects were seen [49]. To summarize, the risk of treatment with vitamin D 30000 IU weekly for eight weeks seem small in the view of published studies. Further, the eligibility criteria of the study will exclude persons of high risk of adverse effects due to treatment. Calcium and 25(OH)D levels will be monitored in the

1. Data Handling and Record Keeping
   1. Case Report Forms
      1. Rules for Completing CRFs

Print should be legibly using a ballpoint pen. All relevant questions should be answered and no empty data blocks should be left. No information is to be recorded outside the data blocks.

If a test/assessment has not been done and will not be available, this is indicated by writing “N/D” (Not Done) in the respective answer field in the CRF. If the question is irrelevant (e.g. is not applicable), this is indicated by writing “N/A” (Not Applicable) in the respective answer field. If the answer is not available, this is indicated by writing “N/K” (Not Known) in the respective answer.

The Investigator or the Investigator’s authorized staff must ensure that all information derived from source documentation is consistent with the source information. By signing the CRFs, the Investigator confirms that the information is complete and correct.

- - 1. Corrections to CRFs

Corrections to the data on the CRFs must only be made by drawing a straight line through the incorrect data and by writing the correct value next to data that has been crossed out. Each correction must be dated, initialed and explained (if necessary) by the Investigator or the Investigator’s authorized staff.

If corrections are made by the Investigator’s authorized staff after the date of the Investigator’s signature, the CRF must be dated and signed again by the Investigator.

- 1. Record Keeping

The Investigator will keep subject notes and other source documents for at least 10 years after the study is reported to the MPA.

The Investigator must archive the documentation pertaining to the trial in an archive after completion of the trial.

1. Insurance

The subjects will be insured by the National Patient Insurance and the National Drug Insurance.

1. Publication Policy

The results from the study is planned to be submitted for publication in an international medical journal and/or presentation at an international congress in 2012 - 2013.

1. Supplements
   1. Amendments

Any changes to this protocol that is not just administrative must be approved by the IEC and/or the MPA before they are implemented.

- 1. Personnel Information

The Investigator will ensure that all personnel involved in the study are well informed about the trial and are able to perform the different tasks regarding the trial, and that this is documented.

1. List of appendices
   1. Appendix A: Declaration of Helsinki
   2. Appendix B: Schedule of investigational visits
   3. Appendix C: IMP labeling
2. References

1. Wild, S., et al., *Global prevalence of diabetes: estimates for the year 2000 and projections for 2030.* Diabetes Care, 2004. **27**(5): p. 1047-53.

2. Stratton, I.M., et al., *Association of glycaemia with macrovascular and microvascular complications of type 2 diabetes (UKPDS 35): prospective observational study.* Bmj, 2000. **321**(7258): p. 405-12.

3. Henriksson, F., et al., *Direct medical costs for patients with type 2 diabetes in Sweden.* J Intern Med, 2000. **248**(5): p. 387-96.

4. Hamman, R.F., *Genetic and environmental determinants of non-insulin-dependent diabetes mellitus (NIDDM).* Diabetes Metab Rev, 1992. **8**(4): p. 287-338.

5. Gillies, C.L., et al., *Pharmacological and lifestyle interventions to prevent or delay type 2 diabetes in people with impaired glucose tolerance: systematic review and meta-analysis.* Bmj, 2007. **334**(7588): p. 299.

6. Ostenson, C.G., *The pathophysiology of type 2 diabetes mellitus: an overview.* Acta Physiol Scand, 2001. **171**(3): p. 241-7.

7. Frayling, T.M., *Genome-wide association studies provide new insights into type 2 diabetes aetiology.* Nat Rev Genet, 2007. **8**(9): p. 657-62.

8. Persson, P.G., et al., *Cigarette smoking, oral moist snuff use and glucose intolerance.* J Intern Med, 2000. **248**(2): p. 103-10.

9. Agardh, E.E., et al., *Work stress and low sense of coherence is associated with type 2 diabetes in middle-aged Swedish women.* Diabetes Care, 2003. **26**(3): p. 719-24.

10. Carlsson, S., et al., *Weight history, glucose intolerance, and insulin levels in middle-aged Swedish men.* Am J Epidemiol, 1998. **148**(6): p. 539-45.

11. Hilding, A., et al., *The impact of family history of diabetes and lifestyle factors on abnormal glucose regulation in middle-aged Swedish men and women.* Diabetologia, 2006. **49**(11): p. 2589-98.

12. Eriksson, A.K., et al., *Psychological distress and risk of pre-diabetes and Type 2 diabetes in a prospective study of Swedish middle-aged men and women.* Diabet Med, 2008. **25**(7): p. 834-42.

13. Pradhan, A.D., et al., *C-reactive protein, interleukin 6, and risk of developing type 2 diabetes mellitus.* Jama, 2001. **286**(3): p. 327-34.

14. Pittas, A.G., et al., *The role of vitamin D and calcium in type 2 diabetes. A systematic review and meta-analysis.* J Clin Endocrinol Metab, 2007. **92**(6): p. 2017-29.

15. Mattila, C., et al., *Serum 25-hydroxyvitamin D concentration and subsequent risk of type 2 diabetes.* Diabetes Care, 2007. **30**(10): p. 2569-70.

16. Forouhi, N.G., et al., *Baseline serum 25-hydroxy vitamin d is predictive of future glycemic status and insulin resistance: the Medical Research Council Ely Prospective Study 1990-2000.* Diabetes, 2008. **57**(10): p. 2619-25.

17. Nilas, L. and C. Christiansen, *Treatment with vitamin D or its analogues does not change body weight or blood glucose level in postmenopausal women.* Int J Obes, 1984. **8**(5): p. 407-11.

18. Pittas, A.G., et al., *The effects of calcium and vitamin D supplementation on blood glucose and markers of inflammation in nondiabetic adults.* Diabetes Care, 2007. **30**(4): p. 980-6.

19. de Boer, I.H., et al., *Calcium plus vitamin D supplementation and the risk of incident diabetes in the Women's Health Initiative.* Diabetes Care, 2008. **31**(4): p. 701-7.

20. Gedik, O. and S. Akalin, *Effects of vitamin D deficiency and repletion on insulin and glucagon secretion in man.* Diabetologia, 1986. **29**(3): p. 142-5.

21. Boucher, B.J., et al., *Glucose intolerance and impairment of insulin secretion in relation to vitamin D deficiency in east London Asians.* Diabetologia, 1995. **38**(10): p. 1239-45.

22. Borissova, A.M., et al., *The effect of vitamin D3 on insulin secretion and peripheral insulin sensitivity in type 2 diabetic patients.* Int J Clin Pract, 2003. **57**(4): p. 258-61.

23. Inomata, S., et al., *Effect of 1 alpha (OH)-vitamin D3 on insulin secretion in diabetes mellitus.* Bone Miner, 1986. **1**(3): p. 187-92.

24. Zofkova, I. and P. Stolba, *Effect of calcitriol and trifluoperazine on glucose stimulated B cell function in healthy humans.* Exp Clin Endocrinol, 1990. **96**(2): p. 185-91.

25. Orwoll, E., M. Riddle, and M. Prince, *Effects of vitamin D on insulin and glucagon secretion in non-insulin-dependent diabetes mellitus.* Am J Clin Nutr, 1994. **59**(5): p. 1083-7.

26. Jorde, R. and Y. Figenschau, *Supplementation with cholecalciferol does not improve glycaemic control in diabetic subjects with normal serum 25-hydroxyvitamin D levels.* Eur J Nutr, 2009. **48**(6): p. 349-54.

27. Nagpal, J., J.N. Pande, and A. Bhartia, *A double-blind, randomized, placebo-controlled trial of the short-term effect of vitamin D3 supplementation on insulin sensitivity in apparently healthy, middle-aged, centrally obese men.* Diabet Med, 2009. **26**(1): p. 19-27.

28. Tai, K., et al., *Glucose tolerance and vitamin D: effects of treating vitamin D deficiency.* Nutrition, 2008. **24**(10): p. 950-6.

29. Lind, L., et al., *Long-term treatment with active vitamin D (alphacalcidol) in middle-aged men with impaired glucose tolerance. Effects on insulin secretion and sensitivity, glucose tolerance and blood pressure.* Diabetes Res, 1989. **11**(3): p. 141-7.

30. Ljunghall, S., et al., *Treatment with one-alpha-hydroxycholecalciferol in middle-aged men with impaired glucose tolerance--a prospective randomized double-blind study.* Acta Med Scand, 1987. **222**(4): p. 361-7.

31. Fliser, D., et al., *No effect of calcitriol on insulin-mediated glucose uptake in healthy subjects.* Eur J Clin Invest, 1997. **27**(7): p. 629-33.

32. Sandhu, M.S., et al., *Circulating concentrations of insulin-like growth factor-I and development of glucose intolerance: a prospective observational study.* Lancet, 2002. **359**(9319): p. 1740-5.

33. Lewitt, M.S., et al., *Insulin-like growth factor-binding protein-1 in the prediction and development of type 2 diabetes in middle-aged Swedish men.* Diabetologia, 2008. **51**(7): p. 1135-45.

34. Hypponen, E., et al., *25-hydroxyvitamin D, IGF-1, and metabolic syndrome at 45 years of age: a cross-sectional study in the 1958 British Birth Cohort.* Diabetes, 2008. **57**(2): p. 298-305.

35. Zittermann, A., *Vitamin D in preventive medicine: are we ignoring the evidence?* Br J Nutr, 2003. **89**(5): p. 552-72.

36. Norman, A.W., et al., *Vitamin D deficiency inhibits pancreatic secretion of insulin.* Science, 1980. **209**(4458): p. 823-5.

37. Bland, R., et al., *Expression of 25-hydroxyvitamin D3-1alpha-hydroxylase in pancreatic islets.* J Steroid Biochem Mol Biol, 2004. **89-90**(1-5): p. 121-5.

38. Simpson, R.U., G.A. Thomas, and A.J. Arnold, *Identification of 1,25-dihydroxyvitamin D3 receptors and activities in muscle.* J Biol Chem, 1985. **260**(15): p. 8882-91.

39. Maestro, B., et al., *Stimulation by 1,25-dihydroxyvitamin D3 of insulin receptor expression and insulin responsiveness for glucose transport in U-937 human promonocytic cells.* Endocr J, 2000. **47**(4): p. 383-91.

40. Chiu, K.C., L.M. Chuang, and C. Yoon, *The vitamin D receptor polymorphism in the translation initiation codon is a risk factor for insulin resistance in glucose tolerant Caucasians.* BMC Med Genet, 2001. **2**: p. 2.

41. Ogunkolade, B.W., et al., *Vitamin D receptor (VDR) mRNA and VDR protein levels in relation to vitamin D status, insulin secretory capacity, and VDR genotype in Bangladeshi Asians.* Diabetes, 2002. **51**(7): p. 2294-300.

42. Ortlepp, J.R., et al., *The vitamin D receptor gene variant and physical activity predicts fasting glucose levels in healthy young men.* Diabet Med, 2003. **20**(6): p. 451-4.

43. Hitman, G.A., et al., *Vitamin D receptor gene polymorphisms influence insulin secretion in Bangladeshi Asians.* Diabetes, 1998. **47**(4): p. 688-90.

44. DeFronzo, R.A., J.D. Tobin, and R. Andres, *Glucose clamp technique: a method for quantifying insulin secretion and resistance.* Am J Physiol, 1979. **237**(3): p. E214-23.

45. Ramos-Zavala, M.G., et al., *Effect of Diacerein on Insulin Secretion and Metabolic Control in Drug-Naive Patients With Type 2 Diabetes: A randomized clinical trial.* Diabetes Care.

46. Hathcock, J.N., et al., *Risk assessment for vitamin D.* Am J Clin Nutr, 2007. **85**(1): p. 6-18.

47. Romagnoli, E., et al., *Short and long-term variations in serum calciotropic hormones after a single very large dose of ergocalciferol (vitamin D2) or cholecalciferol (vitamin D3) in the elderly.* J Clin Endocrinol Metab, 2008. **93**(8): p. 3015-20.

48. Mocanu, V., et al., *Long-term effects of giving nursing home residents bread fortified with 125 microg (5000 IU) vitamin D(3) per daily serving.* Am J Clin Nutr, 2009. **89**(4): p. 1132-7.

49. Amir, E., et al., *A phase 2 trial exploring the effects of high-dose (10,000 IU/day) vitamin D(3) in breast cancer patients with bone metastases.* Cancer, 2010. **116**(2): p. 284-91.

50. Cipriani, C., et al., *Effect of a single oral dose of 600,000 IU of cholecalciferol on serum calciotropic hormones in young subjects with vitamin D deficiency: a prospective intervention study.* J Clin Endocrinol Metab, 2010. **95**(10): p. 4771-7.

51. Sanders, K.M., et al., *Annual high-dose oral vitamin D and falls and fractures in older women: a randomized controlled trial.* Jama, 2010. **303**(18): p. 1815-22.

52. Martineau, A.R., et al., *High-dose vitamin D(3) during intensive-phase antimicrobial treatment of pulmonary tuberculosis: a double-blind randomised controlled trial.* Lancet, 2011. **377**(9761): p. 242-50.

53. von Restorff, C., H.A. Bischoff-Ferrari, and R. Theiler, *High-dose oral vitamin D3 supplementation in rheumatology patients with severe vitamin D3 deficiency.* Bone, 2009. **45**(4): p. 747-9.

1. Signed Agreement of the Trial Protocol

| **Principal Investigator** Henrik Wagner, MD  Dept. of Endocrinology, Metabolism and Diabetes  Karolinska University Hospital |  |  |
| --- | --- | --- |
| Signature |  | Date |
| **Sponsor** Claes Göran Östenson, Prof.,  Dept. of Endocrinology, Metabolism and Diabetes  Karolinska University Hospital |  |  |
| Signature |  | Date |

1. Appendix A: Declaration of Helsinki

**WORLD MEDICAL ASSOCIATION DECLARATION OF HELSINKI**

**Ethical Principles for Medical Research Involving Human Subjects**

Adopted by the 18th WMA General Assembly, Helsinki, Finland, June 1964, and amended by the:

29th WMA General Assembly, Tokyo, Japan, October 1975

35th WMA General Assembly, Venice, Italy, October 1983

41st WMA General Assembly, Hong Kong, September 1989

48th WMA General Assembly, Somerset West, Republic of South Africa, October 1996

52nd WMA General Assembly, Edinburgh, Scotland, October 2000

53rd WMA General Assembly, Washington 2002 (Note of Clarification on paragraph 29 added)

55th WMA General Assembly, Tokyo 2004 (Note of Clarification on Paragraph 30 added)

59th WMA General Assembly, Seoul, October 2008

**A. INTRODUCTION**

1. The World Medical Association (WMA) has developed the Declaration of Helsinki as a

statement of ethical principles for medical research involving human subjects**,** including

research on identifiable human material and data.

The Declaration is intended to be read as a whole and each of its constituent paragraphs

should not be applied without consideration of all other relevant paragraphs.

2. Although the Declaration is addressed primarily to physicians, the WMA encourages

other participants in medical research involving human subjects to adopt these

principles.

3. It is the duty of the physician to promote and safeguard the health of patients, including

those who are involved in medical research. The physician's knowledge and conscience

are dedicated to the fulfilment of this duty.

4. The Declaration of Geneva of the WMA binds the physician with the words, “The

health of my patient will be my first consideration,” and the International Code of

Medical Ethics declares that, “A physician shall act in the patient's best interest when

providing medical care.”

5. Medical progress is based on research that ultimately must include studies involving

human subjects. Populations that are underrepresented in medical research should be

provided appropriate access to participation in research.

6. In medical research involving human subjects, the well-being of the individual research

subject must take precedence over all other interests.

7. The primary purpose of medical research involving human subjects is to understand the

causes, development and effects of diseases and improve preventive, diagnostic and

therapeutic interventions (methods, procedures and treatments). Even the best current

interventions must be evaluated continually through research for their safety,

effectiveness, efficiency, accessibility and quality.

8. In medical practice and in medical research, most interventions involve risks and

burdens.

9. Medical research is subject to ethical standards that promote respect for all human

subjects and protect their health and rights. Some research populations are particularly

vulnerable and need special protection. These include those who cannot give or refuse

consent for themselves and those who may be vulnerable to coercion or undue

influence.

10. Physicians should consider the ethical, legal and regulatory norms and standards for

research involving human subjects in their own countries as well as applicable

international norms and standards. No national or international ethical, legal or

regulatory requirement should reduce or eliminate any of the protections for research

subjects set forth in this Declaration.

**B. PRINCIPLES FOR ALL MEDICAL RESEARCH**

11. It is the duty of physicians who participate in medical research to protect the life, health,

dignity, integrity, right to self-determination, privacy, and confidentiality of personal

information of research subjects.

12. Medical research involving human subjects must conform to generally accepted

scientific principles, be based on a thorough knowledge of the scientific literature, other

relevant sources of information, and adequate laboratory and, as appropriate, animal

experimentation. The welfare of animals used for research must be respected.

13. Appropriate caution must be exercised in the conduct of medical research that may

harm the environment.

14. The design and performance of each research study involving human subjects must be

clearly described in a research protocol. The protocol should contain a statement of the

ethical considerations involved and should indicate how the principles in this

Declaration have been addressed. The protocol should include information regarding

funding, sponsors, institutional affiliations, other potential conflicts of interest,

incentives for subjects and provisions for treating and/or compensating subjects who are

harmed as a consequence of participation in the research study. The protocol should

describe arrangements for post-study access by study subjects to interventions identified

as beneficial in the study or access to other appropriate care or benefits.

15. The research protocol must be submitted for consideration, comment, guidance and

approval to a research ethics committee before the study begins. This committee must

be independent of the researcher, the sponsor and any other undue influence. It must

take into consideration the laws and regulations of the country or countries in which the

research is to be performed as well as applicable international norms and standards but

these must not be allowed to reduce or eliminate any of the protections for research

subjects set forth in this Declaration. The committee must have the right to monitor

ongoing studies. The researcher must provide monitoring information to the committee,

especially information about any serious adverse events. No change to the protocol may

be made without consideration and approval by the committee.

16. Medical research involving human subjects must be conducted only by individuals with

the appropriate scientific training and qualifications. Research on patients or healthy

volunteers requires the supervision of a competent and appropriately qualified physician

or other health care professional. The responsibility for the protection of research

subjects must always rest with the physician or other health care professional and never

the research subjects, even though they have given consent.

17. Medical research involving a disadvantaged or vulnerable population or community is

only justified if the research is responsive to the health needs and priorities of this

population or community and if there is a reasonable likelihood that this population or

community stands to benefit from the results of the research.

18. Every medical research study involving human subjects must be preceded by careful

assessment of predictable risks and burdens to the individuals and communities

involved in the research in comparison with foreseeable benefits to them and to other

individuals or communities affected by the condition under investigation.

19. Every clinical trial must be registered in a publicly accessible database before

recruitment of the first subject.

20. Physicians may not participate in a research study involving human subjects unless they

are confident that the risks involved have been adequately assessed and can be

satisfactorily managed. Physicians must immediately stop a study when the risks are

found to outweigh the potential benefits or when there is conclusive proof of positive

and beneficial results.

21. Medical research involving human subjects may only be conducted if the importance of

the objective outweighs the inherent risks and burdens to the research subjects.

22. Participation by competent individuals as subjects in medical research must be

voluntary. Although it may be appropriate to consult family members or community

leaders, no competent individual may be enrolled in a research study unless he or she

freely agrees.

23. Every precaution must be taken to protect the privacy of research subjects and the

confidentiality of their personal information and to minimize the impact of the study on

their physical, mental and social integrity.

24. In medical research involving competent human subjects, each potential subject must be

adequately informed of the aims, methods, sources of funding, any possible conflicts of

interest, institutional affiliations of the researcher, the anticipated benefits and potential

risks of the study and the discomfort it may entail, and any other relevant aspects of the

study. The potential subject must be informed of the right to refuse to participate in the

study or to withdraw consent to participate at any time without reprisal. Special

attention should be given to the specific information needs of individual potential

subjects as well as to the methods used to deliver the information. After ensuring that

the potential subject has understood the information, the physician or another

appropriately qualified individual must then seek the potential subject’s freely-given

informed consent, preferably in writing. If the consent cannot be expressed in writing,

the non-written consent must be formally documented and witnessed.

25. For medical research using identifiable human material or data, physicians must

normally seek consent for the collection, analysis, storage and/or reuse. There may be

situations where consent would be impossible or impractical to obtain for such research

or would pose a threat to the validity of the research. In such situations the research may

be done only after consideration and approval of a research ethics committee.

26. When seeking informed consent for participation in a research study the physician

should be particularly cautious if the potential subject is in a dependent relationship

with the physician or may consent under duress. In such situations the informed consent

should be sought by an appropriately qualified individual who is completely

independent of this relationship.

27. For a potential research subject who is incompetent, the physician must seek informed

consent from the legally authorized representative. These individuals must not be

included in a research study that has no likelihood of benefit for them unless it is

intended to promote the health of the population represented by the potential subject,

the research cannot instead be performed with competent persons, and the research

entails only minimal risk and minimal burden.

28. When a potential research subject who is deemed incompetent is able to give assent to

decisions about participation in research, the physician must seek that assent in addition

to the consent of the legally authorized representative. The potential subject’s dissent

should be respected.

29. Research involving subjects who are physically or mentally incapable of giving

consent, for example, unconscious patients, may be done only if the physical or mental

condition that prevents giving informed consent is a necessary characteristic of the

research population. In such circumstances the physician should seek informed consent

from the legally authorized representative. If no such representative is available and if

the research cannot be delayed, the study may proceed without informed consent

provided that the specific reasons for involving subjects with a condition that renders

them unable to give informed consent have been stated in the research protocol and the

study has been approved by a research ethics committee. Consent to remain in the

research should be obtained as soon as possible from the subject or a legally authorized

representative.

30. Authors, editors and publishers all have ethical obligations with regard to the

publication of the results of research. Authors have a duty to make publicly available

the results of their research on human subjects and are accountable for the completeness

and accuracy of their reports. They should adhere to accepted guidelines for ethical

reporting. Negative and inconclusive as well as positive results should be published or

otherwise made publicly available. Sources of funding, institutional affiliations and

conflicts of interest should be declared in the publication. Reports of research not in

accordance with the principles of this Declaration should not be accepted for

publication.

**C. ADDITIONAL PRINCIPLES FOR MEDICAL RESEARCH COMBINED WITH**

**MEDICAL CARE**

31. The physician may combine medical research with medical care only to the extent that

the research is justified by its potential preventive, diagnostic or therapeutic value and if

the physician has good reason to believe that participation in the research study will not

adversely affect the health of the patients who serve as research subjects.

32. The benefits, risks, burdens and effectiveness of a new intervention must be tested

against those of the best current proven intervention, except in the following

circumstances:

• The use of placebo, or no treatment, is acceptable in studies where no current

proven intervention exists; or

• Where for compelling and scientifically sound methodological reasons the use of

placebo is necessary to determine the efficacy or safety of an intervention and the

patients who receive placebo or no treatment will not be subject to any risk of

serious or irreversible harm. Extreme care must be taken to avoid abuse of this

option.

33. At the conclusion of the study, patients entered into the study are entitled to be

informed about the outcome of the study and to share any benefits that result from it, for

example, access to interventions identified as beneficial in the study or to other

appropriate care or benefits.

34. The physician must fully inform the patient which aspects of the care are related to the

research. The refusal of a patient to participate in a study or the patient’s decision to

withdraw from the study must never interfere with the patient-physician relationship.

35. In the treatment of a patient, where proven interventions do not exist or have been

ineffective, the physician, after seeking expert advice, with informed consent from the

patient or a legally authorized representative, may use an unproven intervention if in the

physician's judgement it offers hope of saving life, re-establishing health or alleviating

suffering. Where possible, this intervention should be made the object of research,

designed to evaluate its safety and efficacy. In all cases, new information should be

recorded and, where appropriate, made publicly available.

1. Appendix B: Schedule of investigational visits

1. Appendix C: IMP labelling

[**För klinisk prövning**](https://apoteket.se/rd/d/10)

Studie: **EVIDENS DvitamininDM2_v1_110815.doc *(***Eudra-CT nr: 2010-024487-18)

Randomiserings-nr: ……………......... Pat-initialer: ……………..

**Dosering: 1,5 ml intages oralt en gång per vecka.**

Vigantol Oil/placebo, 20 000 IU/ml, lösning, orala droppar, 10 ml.

Sats-nr: ........................ Förvaras ljusskyddat i förpackningskartong.

Utgångsdatum: .............................. Hållbarhet 6 mån efter att flaskan öppnats.

Förvaras utom syn- och räckhåll för barn.

Sponsor: Claes Göran Östenson Ansvarig prövare: Henrik Wagner

Endokrinkliniken, Karolinska Universitetssjukhuset, Solna

171 76 Stockholm, tel: 08-517 700 00

1. Trial-related activities are any procedure that would not have been performed during normal management of the subject. [↑](#footnote-ref-2)
